# Discovery of MicroRNAs and Their Target Genes Related to Drought in *Paulownia* “Yuza 1” by High-Throughput Sequencing

**DOI:** 10.1155/2017/3674682

**Published:** 2017-06-11

**Authors:** Minjie Deng, Yabing Cao, Zhenli Zhao, Lu Yang, Yanfang Zhang, Yanpeng Dong, Guoqiang Fan

**Affiliations:** ^1^Institute of Paulownia, Henan Agricultural University, Zhengzhou, Henan 450002, China; ^2^College of Forestry, Henan Agricultural University, Zhengzhou, Henan 450002, China

## Abstract

Understanding the role of miRNAs in regulating the molecular mechanisms responsive to drought stress was studied in *Paulownia* “yuza 1.” Two small RNA libraries and two degradome libraries were, respectively, constructed and sequenced in order to detect miRNAs and their target genes associated with drought stress. A total of 107 miRNAs and 42 putative target genes were identified in this study. Among them, 77 miRNAs were differentially expressed between drought-treated *Paulownia* “yuza 1” and the control (60 downregulated and 17 upregulated). The predicted target genes were annotated using the GO, KEGG, and Nr databases. According to the functional classification of the target genes, *Paulownia* “yuza 1” may respond to drought stress via plant hormone signal transduction, photosynthesis, and osmotic adjustment. Furthermore, the expression levels of seven miRNAs (ptf-miR157b, ptf-miR159b, ptf-miR398a, ptf-miR9726a, ptf-M2153, ptf-M2218, and ptf-M24a) and their corresponding target genes were validated by quantitative real-time PCR. The results provide relevant information for understanding the molecular mechanism of *Paulownia* resistance to drought and reference data for researching drought resistance of other trees.

## 1. Introduction

*Paulownia* is a genus that contains indigenous tree species in China with a long history of cultivation over 2000 years and now spreads around the world [1]. Because of its excellent material properties, *Paulownia* has great economic value (plywood, string instruments, and paper making), ecological and humanistic value (ornamental, road virescence and afforestation, windbreak, and sand fixation), and social value (national defense construction and medications) [2, 3]. *Paulownia* trees are deciduous and fast growing and can be planted under water-deficit and high-salt conditions. *Paulownia* “yuza 1,” a hybrid clone of *Paulownia tomentosa* and *P. fortunei*, has inherited the fine characteristics of its parentage, in particular its prominent ability to grow in infertile soil [4].

Environmental stresses adversely influence plant growth and development. Water deficit, more than any other factor, was reported to be responsible for reduced production worldwide. In recent years, the arid areas have become more and more expansive, and much attention has been focused on drought, which is the most destructive impact factor for plant development [5, 6]. Some plants have evolved a complex and precise regulatory network to counter drought stress, such as reducing the leaf area by lowering leaf growth rate, increasing the fall off rate of old leaves, and closing the plant stomata by the increase of abscisic acid (ABA) in the guard cells to conserve water, which ultimately lead to an inhibition of many biological processes, such as respiration, photosynthetic activity, and CO_2_ assimilation [7]. Besides ABA, drought stress could also disturb the balance of other endogenous phytohormones, such as auxin, cytokinin, gibberellin (GA), jasmonate, and brassinosteroid. Previous studies demonstrate that the decrease of GA levels and signaling may be associated with plant growth restriction when plants are exposed to drought or salinity condition [8]. All the processes above are involve in gene expression changes which result from transcriptional and posttranscriptional regulation.

MicroRNAs (miRNAs) are small endogenous RNAs that are 20–24 nt long. Primary miRNAs are transcribed from nonprotein-coding genes, processed by cellular nucleases into precursor miRNAs, which are cleaved to generate the mature miRNAs. The mature miRNAs are assembled into the RNA-induced silencing complex (RISC), which regulates gene expressions at the posttranscriptional level [9]. In plants, miRNA-mediated gene regulation has involved in numerous processes, including developmental processes, hormone regulation, nutrient homeostasis, and stress response [10–13]. Genetic information can help in understanding complicated gene regulation networks that work under environmental stresses [14]. Recently, many miRNAs related to stresses were identified in woody plants, including *Populus*, peach, *Citrus*, and *Malus* [15–19]. In *Populus*, two miRNAs, miR164 and miR473, and their potential targets that expressed differentially under drought stress were considered related to the drought tolerance mechanism [20]. In peach, miRNA160 which was known to target auxin response factor (ARF) was upregulated in peach roots [16]. Besides, the GO and KEGG analysis of the targets for miRNAs in *Citrus junos* showed that a number of metabolic, physiological, and hormonal responses were involved in drought stress, including carbohydrate metabolism, plant hormone signal transduction, and protein phosphorylation [21]. GO functional classification and KEGG analysis showed that miRNAs might play roles in response to drought and salinity stresses through targeting a series of stress-related genes in cotton [22]. Together, all these findings manifested the role of miRNAs and their potential target genes in plants under drought condition. However, as little molecular genetics research focused on *Paulownia* has been reported, the mechanism of drought resistance in *Paulownia* “yuza 1” is still unclear caused by a lack of the full *Paulownia* genome information. Therefore, small RNA libraries and degradome libraries of drought-treated *Paulownia* “yuza 1” plants and the controls were, respectively, constructed, sequenced, and analyzed to investigate the drought responsive mechanism in *Paulownia* “yuza 1” at posttranscriptional level. We speculated that microRNAs may play an essential role in *Paulownia* “yuza 1” responsive to drought stress via plant hormone signal transduction, photosynthesis, and osmotic adjustment. The results will contribute to understanding the molecular mechanism associated with the drought resistance response in *Paulownia* “yuza 1” and provide reference data for researching drought resistance in other trees and for breeding drought-tolerant varieties.

## 2. Materials and Methods

### 2.1. Plant Material and Treatment

Uniformly grown 30-day diploid *Paulownia* “yuza 1” (PTF2) seedlings obtained by tissue culture from the Institute of Paulownia, Henan Agricultural University, Zhengzhou, Henan Province, China, were chosen as the experimental material. Consistently grown plantlets were collected and planted in plastic pots with garden soil (main components: composting plant straw, peat, perlite, soybean dregs, trace elements, and vermiculite). The plastic pots were 20 cm in diameter at the bottom and 20 cm deep, one for each plant. The sample plants were placed randomly in the nursery with a rain shelter. After 50 days, the plants were subjected to drought stress. Twelve plants of PTF2 were obtained and evenly divided into two groups. The control group (PTF2W) was watered to field capacity daily (with 75% relative soil water content). The treated group (PTF2T) was subjected to severe drought stress (25% relative soil water content) using a water-controlled experiment according to the method of Zhang et al. [23]. The soil water content was measured by weighing at 18:00 PM every day. Then each pot was supplemented with water to maintain the relative soil water content at 75% (control group) and 25% (treated group). After 20 days, three individuals with coincident growth conditions were selected from each group (PTF2W and PTF2T) for collecting leaves. The leaves (the second pair from the apex) were harvested and mixed in each of the groups. The harvested leaves were frozen in liquid nitrogen immediately and stored at −80°C.

### 2.2. Construction and Sequencing of the sRNA Libraries

Approximately 4 mg leaves from PTF2W and PTF2T were used to extract total RNA with TRIzol reagent (Invitrogen, Carlsbad, CA). The integrity of RNA was checked using a 2001 Bioanalyzer (Agilent Technologies Inc., Santa Clara, CA). Extracted RNA was separated into fragments of different sizes by PAGE, and fragments with 18–30 nt were cut out and reclaimed. 5′ and 3′ adapters were ligated to the reclaimed fragments. The products were used as templates for reverse transcription to obtain double-stranded cDNA, which was then amplified by 12 cycles on PCR, according to procedures. The PCR products were recycled and purified by PAGE, then dissolved in ethidium bromide (EB) solution. Fragments with 140–160 bp were selected to establish the two sRNA libraries (PTF2W and PTF2T) for sequencing on an Illumina HiSeq™ 2000 platform (Illumina, San Diego, CA, USA).

### 2.3. Identification of miRNAs

The raw reads obtained by Illumina sequencing were filtered to remove sequences that contained adapters, insertions, poly (A) tails, and reads smaller than 18 nt. The quality and length distribution of the resultant clean reads were analyzed, and the clean reads were matched with *Paulownia* “yuza 1” unigenes using SOAP. To identify conserved miRNAs, the reads were mapped to plant sequences in miRBase (Release 21.0) (http://microrna.anger.ac.uk/sequences) using blastall owing no more than two mismatches. The clean reads were also aligned to sequences in the GenBank (http://www.ncbi.nlm.nih.gov/) and Rfam (http://www.sanger.ac.uk/software/Rfam) databases using Blast (http://www.ncbi.nlm.nih.gov/staff/tao/URLAPI/blastall/). The reads that mapped to rRNA, scRNA, snoRNA, snRNA, and tRNA sequences were removed. The sRNAs that were unannotated and matched exon antisense strands, introns, and intergenic region were analyzed by Mireap to identify novel miRNAs (https://sourceforge.net/projects/mireap/), and the software Mfold (http://mfold.rna.albany.edu/?q0mfold) was used to predict secondary structures of novel miRNAs. The criteria used to predict novel miRNAs have been described previously [24].

### 2.4. Differential Expression Analysis of miRNAs in Drought-Stressed *Paulownia* “Yuza 1”

The expressions of the conserved and novel miRNAs in the two libraries were calculated, and the differences in their expression levels were determined by comparing the two libraries. The numbers of miRNAs in the two libraries were normalized to one million reads. Fold changes in miRNA expression between PTF2T and PTF2W and *P* values were calculated based on the normalized data (normalized expression = actual miRNA count/total count of clean reads × 1,000,000). *P* value ≤ 0.05 and fold changes ≥ 1 or ≤−1 were used to identify differentially expressed miRNAs. *P* value ≤ 0.01 and fold changes ≥ 1 or ≤−1 were used to identify significantly differentially expressed miRNAs. Afterwards, fold changes and *P* values of miRNAs were calculated based on the normalized data.

Fold change = log_2_ (normalized miRNA reads in PTF2T/normalized miRNA reads in PTF2W).

*P* value is

(1) $$\begin{array}{c} P\left(x\left|y\right.\right)=\left(\frac{N_{2}}{N_{1}}\right)\frac{\left(x+y\right)!}{x!y!{\left(1+\left(N_{2}/N_{1}\right)\right)}^{\left(x+y+1\right)}},\\ C\left(y\leq y_{\min}\left|x\right.\right)=\overset{y\leq y_{\min}}{\underset{y=0}{\sum }}P\left(y\left|x\right.\right),\\ D\left(y\geq y_{\max}\left|x\right.\right)=\overset{\infty }{\underset{y\geq y_{\max}}{\sum }}P\left(y\left|x\right.\right), \end{array}$$

where *N*_1_ and *N*_2_ represent the total number of clean reads, *x* and *y* represent the number of miRNAs surveyed in PTF2T and PTF2W, respectively, and *C* and *D* can be regarded as the probability discrete distribution of the *P* value inspection.

### 2.5. Prediction of miRNA Target Genes

To determine the target genes of the miRNAs, two degradome libraries (PTF2W and PTF2T) were constructed and sequenced as described previously [25]. A data cleaning analysis of the 49 nt sequence tags was performed to obtain credible clean tags. Data quality and length distribution of the clean tags were analyzed, and the clean tags were mapped to *Paulownia* “yuza 1” unigenes using SOAP. The clean tags were searched against GenBank and Rfam databases for annotation. Tags annotated as noncoding RNA (rRNA, tRNA, scRNA, snRNA, and snoRNA) and those containing poly (N) sequences were removed. The annotations were ranked using Rfam > GenBank > poly (N). Tags that had no annotation were mapped to the reference unigene set of *Paulownia* “yuza 1” to predict the possible cleavage sites for mRNA-miRNA binding. Finally, the predicted targets were matched to the protein databases, including NCBI nonredundant protein sequence database (Nr), NCBI nucleotide database (Nt), and Swiss-Prot database using BLAST with a cutoff *E* value of 1.0 × 10^−5^. Gene Ontology (GO) functional annotations were performed with the use of the software BLAST2GO according to Nr annotations. Additionally, Kyoto Encyclopedia of Genes and Genomes (KEGG) pathway analysis used blastall at *E* values ≤ 10^−5^.

### 2.6. Quantitative Real-Time PCR

To validate the differentially expressed miRNAs and their corresponding target unigenes, quantitative real-time PCR (qRT-PCR) was carried out. The miRNAs were chosen randomly along with their target unigenes. For the miRNAs, the forward primers were designed based on the mature miRNA sequences, the reverse primers were the frequently used reverse primers, and U6 was used as the endogenous control. First-strand cDNAs were synthesized using a Mir-X™ miRNA First-Strand Synthesis Kit (TaKaRa Biotechnology, Dalian, China), and amplifications were performed on a CFX96™ Real-Time System (Bio-Rad, Hercules, CA, USA). For the miRNA targets, the primers were designed using Beacon Designer version 7.7 (Premier Biosoft International Ltd., Palo Alto, CA, USA), with 18S rRNA as the endogenous reference gene (see Supplementary Table S1 and Table S2 available online at https://doi.org/10.1155/2017/3674682). The PCRs were performed as 50°C for 3 min, 95°C for 5 min, 40 cycles of 95°C for 15 s, 55°C for 30 s, and 40°C for 10 min. The 2^−ΔΔCt^ method was used to calculate the relative expression levels of the miRNAs and their targets as described previously [26]. Each gene had three biological replicates. An independent *t*-test was performed using SPSS 19.0 (IBM Corp., Armonk, NY).

## 3. Results

### 3.1. Analysis of Two *Paulownia* “Yuza 1” Small RNA Libraries

**Figure 1:**
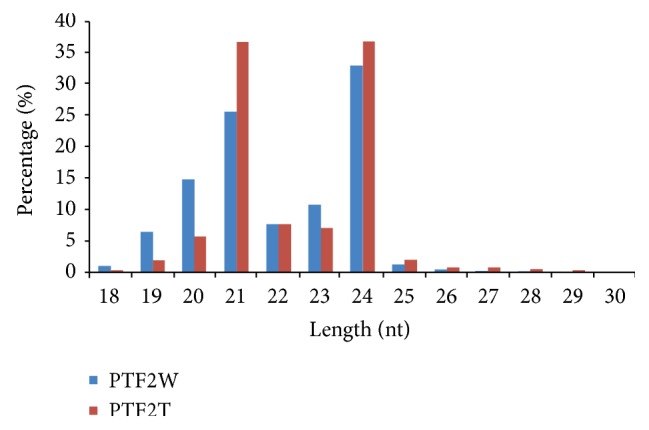
Length distribution of sRNAs in *Paulownia* “yuza 1” PTF2W (control) and PTF2T (severe stress).

High-throughput sequencing generated 23,221,177 raw reads from the two libraries. After eliminating low-quality reads, 23,168,277 high-quality reads were obtained. Subsequently, 23,040,002 clean reads (99.45% of the high-quality reads) were acquired by removing the reads that contained adaptors and poly (A) tails. Length distribution analysis of clean reads revealed two major peaks at 24 and 21 nt (Figure 1). The clean reads were aligned to the *Paulownia* “yuza 1” unigene dataset and also mapped to the GenBank and Rfam databases for annotation. Finally, the unclassified 6,212,092 unique sRNAs were used for predicting potential novel miRNAs (Table 1).

### 3.2. Identification of Conserved and Novel miRNAs

The unique sRNA sequences were searched against known plant miRNA sequences in miRBase 21.0 with two or fewer mismatches to identify conserved miRNAs. A total of 32 conserved miRNAs were identified, and 20 of these miRNAs had miRNA^∗^ sequences (Table 1, Table S3). Among the conserved miRNAs, the 21 nt long miRNAs were the most abundant, followed by the 22 nt miRNAs. The expression levels of the miRNAs determined from the sRNA-seq data ranged widely from 0 to 1,677,167. Except for ptf-miR9726, all the other conserved miRNAs were expressed in both libraries. Ptf-miR166 was the most highly expressed, accounting for 59.27%, while ptf-miR9726 showed the lowest expression.

The 6,212,092 unclassified unique sRNAs that did not find matches in any of the searched databases were used to predict potential novel miRNAs. Seventy-five candidate novel miRNAs were identified. Among them, 12 had miRNA^∗^ sequences (Table 1, Table S2), and 61.33% were 21 nt long miRNAs, followed by those that were 23 nt long. Except for five miRNAs (ptf-M1955, ptf-M2153, ptf-M2358, ptf-M4158, and ptf-M625), the expression levels of the other novel miRNAs were lower than the expression levels of the conserved miRNAs. The average length of the novel precursor miRNAs was 150 nt, and the average minimum free energy of the predicted hairpin structures was −45.86 kcal/mol. The mature miRNAs were localized in the 5′ and 3′ arms of the stem-loop structures; 41 novel miRNAs were located in the 3′ arm, and 34 were in the 5′ arm.

### 3.3. Differentially Expressed miRNAs in Drought-Stressed *Paulownia* “Yuza 1”

miRNAs with *P* value ≤ 0.05 and fold changes ≥ 1 or ≤−1 were determined as up- or down-regulated miRNAs. miRNAs with *P* value ≤ 0.01 and fold changes ≥ 1 or ≤−1 were significantly up- or down-regulated. Seventy-seven differentially expressed miRNAs were found in the PTF2T versus PTF2W comparison (60 were downregulated and 17 were upregulated); 60 of them were significantly differentially expressed (44 significantly downregulated and 16 significantly upregulated) (Table S4). Some of these differentially expressed miRNAs are likely to be involved in the response of *Paulownia* “yuza 1” to drought stress.

### 3.4. Identified Target Genes of miRNAs

**Figure 2:**
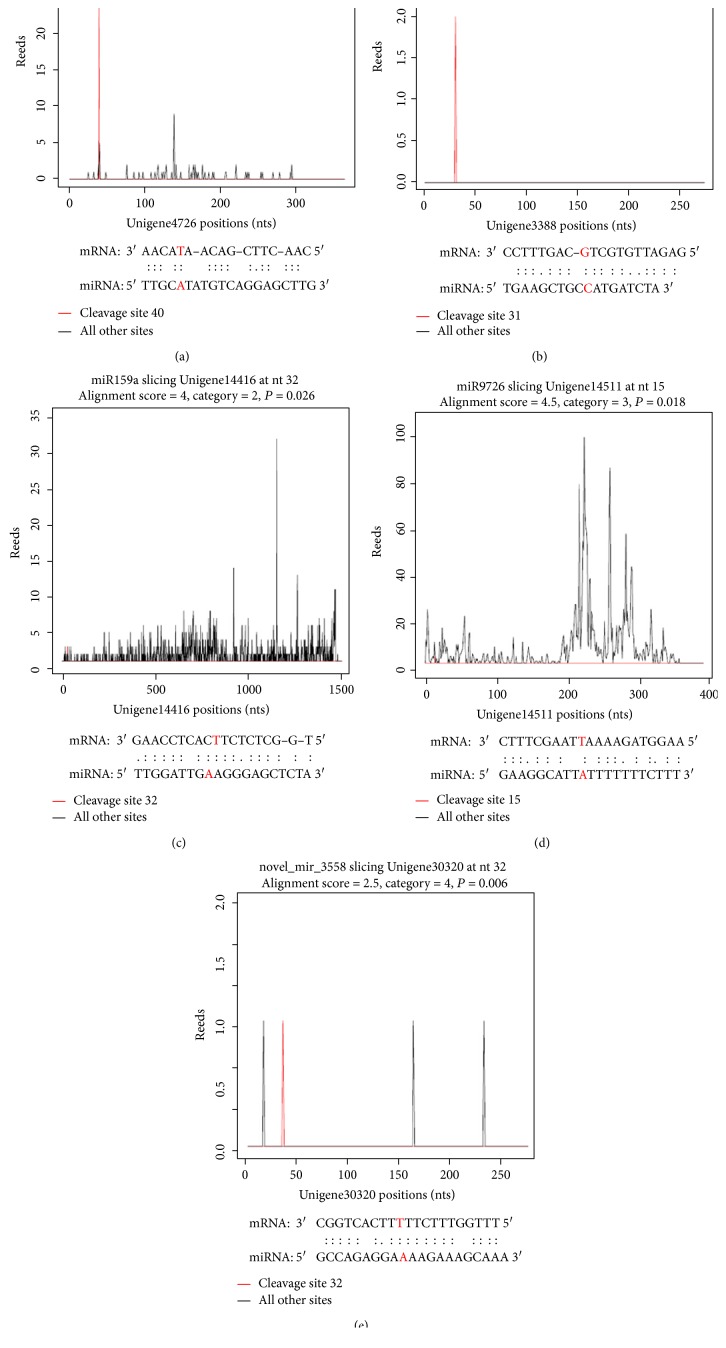
Target plots (t-plots) of miRNA targets in *Paulownia* “yuza 1” PTF2W (control) and PTF2T (severe stress). (a) Category 0; (b) category 1; (c) category 2; (d) category 3; (e) category 4.

To reveal the possible roles of miRNAs in the drought stress response, we used degradome sequencing to identify miRNA target genes (Table 2). As a result, we found 42 target genes (with 47 cleavage sites) that were predicted to be regulated by seven conserved miRNA families and ten novel miRNAs families (Table S4). And these target genes could be divided into five classes (categories 0, 1, 2, 3, and 4) based on specified protocols (Figure 2). In all, one target (one cleavage site) was assigned to category 0, one target (one cleavage site) was assigned to category 1, nine targets (ten cleavage sites) were assigned to category 2, one target (one cleavage site) was assigned to category 3, and 31 targets (34 cleavage sites) belonged to category 4.

### 3.5. Functional Analysis of the Target Genes

**Figure 3:**
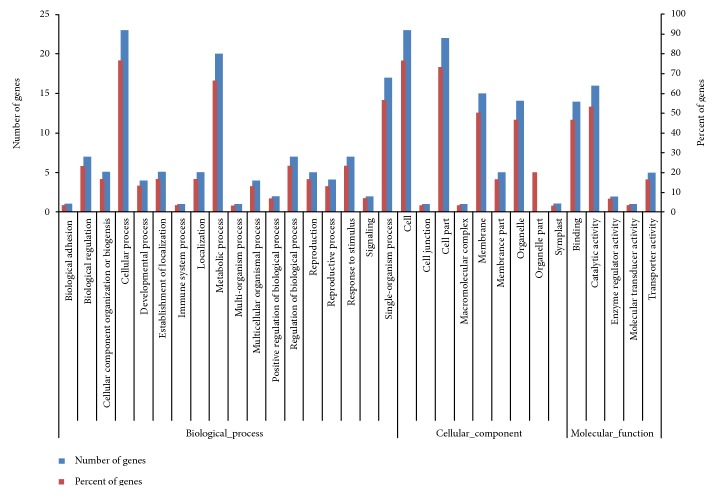
GO classification of putative miRNA targets in *Paulownia* “yuza 1” PTF2W (control) and PTF2T (severe stress).

The target genes were annotated by searching against Nr, Nt, and Swiss-Prot using BLAST (Table S4). To further understand the function of the target genes for miRNAs, functional classification and pathway analysis of these 42 putative target genes were performed using the GO and KEGG databases (Figure 3, Table S5). A total of 24, 25, and 20 targets were, respectively, assigned to biological processes, cellular components, and molecular functions in the GO database. In the category *biological processes*, seven targets (CL1233.Contig1, CL4115.Contig1, Unigene2124, CL3316.Contig6, Unigene1667, CL10424.Contig2, and CL5086.Contig2) were classified to the group *response to stimulus*; and two targets (Unigene14416 and CL1233.Contig1) were classified to the group *positive regulation of biological process*. Five targets (Unigene14416, CL3316.Contig6, Unigene1667, Unigene24393, and CL1482.Contig2) belonged to the group *transporter activity* in the molecular function category. In the category *cellular component*, *cell* and *cell part* were the most highly represented GO terms. The KEGG analysis predicted that 24 targets were annotated and involved in 28 metabolic pathways, including plant hormone signal transduction, ABC transporters, and photosynthesis (Table S5).

Among the targets of upregulated miRNAs, the most targets were involved in *cellular process* and *single-organism process* under *biological processes* category; *cell* and *cell part* were the most highly represented GO terms under *cellular component* category; and *binding* was the highly represented under *molecular function* category. Among the targets of downregulated miRNAs, many targets took part in *response to stimulus*, *regulation of biological process*, and *signaling* under *biological processes* category; *cell* and *cell part* were the most highly represented GO terms under *cellular component* category; and many targets were related to *catalytic activity* and *transporter activity* under *molecular function* category. These above findings were consistent with previous studies [27].

### 3.6. Validation of miRNAs and Their Target Genes by qRT-PCR

**Figure 4:**
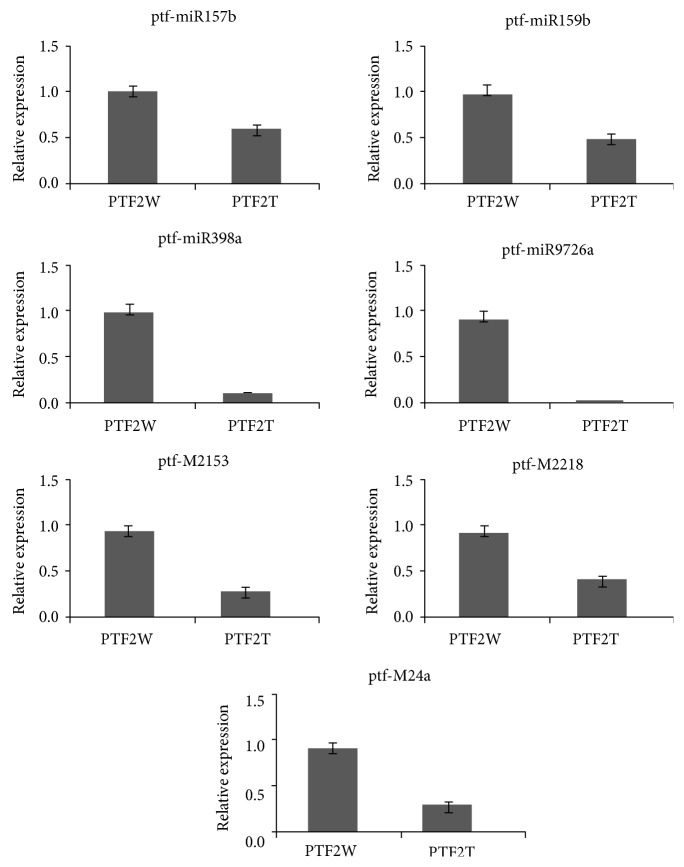
Relative expression levels of seven miRNAs in *Paulownia* “yuza 1” PTF2W (control) and PTF2T (severe stress). Error bars represent ±SD (*n* = 3). The expression levels of miRNAs were normalized to U6. The normalized miRNA levels in the PTF2W were uniformly set to 1. Independent *t*-test had been done and all miRNAs significantly expressed (*P* < 0.05).

**Figure 5:**
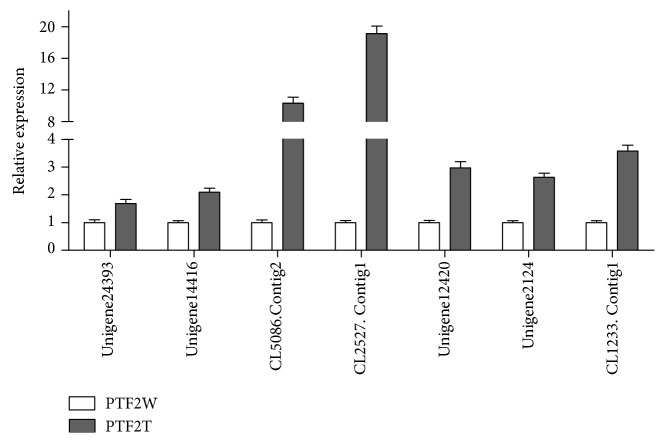
Relative expression levels of putative miRNA targets in *Paulownia* “yuza 1” PTF2W (control) and PTF2T (severe stress). Error bars represent ±SD (*n* = 3). The expression levels of putative target genes were normalized to 18S rRNA. The normalized miRNA levels in the PTF2W were uniformly set to 1. Independent *t*-test had been done and all targets significantly expressed (*P* < 0.05).

To validate the expression results obtained from the sRNA-seq data, seven miRNAs (ptf-miR157b, ptf-miR159b, ptf-miR398a, ptf-miR9726a, ptf-M2153, ptf-M2218, and ptf-M24a) and their target genes (Unigene24393, Unigene14416, CL5086.Contig2, CL2527.Contig1, Unigene12420, Unigene2124, and CL1233.Contig1) were selected randomly for qRT-PCR analysis. The expression levels of the miRNAs obtained by PCR were consistent with those calculated from the sRNA-seq data (Figure 4). The expression levels of the target genes (Figure 5) obtained by qRT-PCR tended to be opposite to those for the miRNAs.

## 4. Discussion

In herbaceous and woody plants, some conserved miRNAs have been reported to be involved in drought resistance. Previous studies have demonstrated that the miR156, which predicted to target the transcription factor SPB12, was downregulated in *P. australis*, *Ammopiptanthus mongolicus*, *Populus tomentosa*, and *Gossypium hirsutum* under drought stress [22, 27–29]. According to the degradome sequencing results in our study, the ptf-miR157a/b was confirmed to target the ABC transporter B family member 19 (ABCB19), which plays an important role in basipetally long-distance auxin transport from the seedling apex to roots, resulting in auxin accumulation to increase formation of adventitious roots [30, 31]. In *Arabidopsis*, overexpression of *ABCB19* led to the accumulation of ABCB19 and increased the adventitious root formation [32, 33]. Both lateral and adventitious roots play significant roles in plant growth and development by providing an efficient network for water and nutrient uptake, as well as by anchoring plants. It has been reported that ABCB19 can enhance gravitropism of plant roots [30, 34], which is conducive to deep rooting. The ptf-miR159a and ptf-miR159b were another two downregulated drought-related miRNAs, which were predicted to target the ABC transporter I family. It has been reported that ABC transporters serve as ATP-dependent pumps and ion channel regulators [35]. In a previous study, we identified a protein belonging to the ABC transporter I family that was specifically induced by salt stress [36]. In the present study, the two miRNAs (ptf-miR159a and ptf-miR159b) targeting ABC transporters were downregulated under drought stress, which is coincident with the results of the previous study. Hence, we speculated that ABC transporters may be positive regulators, which can enhance the tolerance of plants to drought stress.

In plants, immunophilins can prevent injury from environmental stresses. Cytochromes (CYPs) are immunophilins, and several CYP family members had been discovered in model plants such as *A. thaliana*, *Oryza sativa*, *P. trichocarpa*, *Morus notabilis*, and *Carica papaya* [37–41]. In rice, *OsCYP20-2* was shown to take part in the abiotic stress response, and overexpression of *OsCYP18-2* by interacting with ski-interacting protein (SKIP) can enhance drought tolerance in rice and *Arabidopsis* [42]. Additionally, SKIP was also found to participate in splicing precursor mRNA in *Arabidopsis* [43], and the *cis*- and *trans*-isomerase peptidyl-prolyl isomerase-like 1 is recruited by SKIP into the spliceosome where precursor mRNA splicing occurs [44], suggesting that overexpression of *OsCYP18-2* affected the expression of alternative splicing variants of stress-related genes by SKIP under drought condition [42]. Cyclophilin is suggested to be an unspecific stress-responsive protein in plants and plays a role in the defense response, and it was upregulated in potato and tobacco under stresses [45, 46]. In our study, cyclophilin-like protein was encoded by CL2527.Contig1, a target gene of ptf-miR9726, which was downregulated under drought stress. Accordingly, under drought condition, CL2527.Contig1 may be overexpressed, which is in accordance with the results in rice and *Arabidopsis*. Thus, we consider that ptf-miR9726 and its corresponding target CL2527.Contig1 are involved in resistance to drought stress in *Paulownia* “yuza 1.”

In this present study, we also found that some potential target genes of novel miRNAs were predicted to be involved in drought resistance, especially two targets CL1233.Contig1 and Unigene4726 of ptf-M24a/b, which were suggested to encode protein kinases and KSL proteins, respectively. It has been proved that protein kinases play the significant roles in ionic and osmotic homeostasis signal pathways, detoxification response pathways, and stress responses pathways in plants under drought stress condition [47–49]. The protein kinase Pto-interacting protein 1 (Pti1), which has a specific N-terminalis highly conserved in many plants, was involved in response to stimulus [50]. N-terminal myristoylation of Pti1 is a posttranslational modification, which plays a key role in signal transduction in plant responses to abiotic stress [51]. For example, the salt tolerance gene *SOS3* of *Arabidopsis* encodes a calcium-binding protein that was found to contain an N-myristoylation sequence [52], which was shown to play an essential role in salt tolerance in *Arabidopsis* [53]. Recently, Li et al. found that protein kinase Pti-1 was dramatically induced by ABA and triggered the stress-responsive mechanisms to maintain the homeostasis and repair the damaged membranes in maize under drought stress [54]. Besides Pti1, kaurene synthase-like (KSL) proteins have also been associated with drought. Kaurene synthase encoding genes that contribute to the biosynthesis of diterpenoids, especially gibberellic acid (GA), were identified in the rice genome [55, 56]. It had been proposed that in rice, *OsKSL2* was involved in GA biosynthesis [57]. Verma et al. suggested that ABA and GA showed an antagonistic relationship under the abiotic stress conditions [58]. In addition, the more tolerance of dehydration, salinity, heat, and cold in the moderate GA-deficient rice mutant than the nontransformed controls were observed in rice species [59]. In our study, the two targets of ptf-M24a/b, CL1233.Contig1 and Unigene4726, were downregulated under drought stress. Thus, we inferred that ptf-M24a/b of *Paulownia* “yuza 1” may play a key role in resisting drought stress.

Previous studies showed that moderate or severe drought stress could significantly reduce the function and activities of photosystem II (PSII) reaction system [60], which mainly consists of the D1 and D2 proteins. The degradation and synthesis of D1 play key roles in maintaining the stability of the PSII reaction center. Normally, the degradation rate of D1 is lower than the synthetic rate; however, when plants are exposed to bright light or environmental stresses, the degradation rate of D1 is higher than the synthetic rate, which results in the destruction of the PSII reaction center. In *Arabidopsis*, GLK2, a member of the Myb transcription factor family, was reported to play a positive role in the development and maintenance of chloroplasts. Under drought stress, GLK2 promotes the synthesis of the D1 protein to maintain the stability of the PSII reaction center [61, 62]. In this study, the Unigene259 and CL1451.Contig3 genes were annotated as encoding PSII reaction center proteins and the GLK transcription factor, which are involved in the photosynthesis and plant hormone signal transduction and targeted by ptf-M1358 and ptf-M2218, respectively. Therefore, we hypothesized that the downregulated miRNAs ptf-M1358 and ptf-M2218 play important roles in response to drought stress through their negatively expressed corresponding target genes PSII reaction center proteins and the GLK transcription factor. As a result, *Paulownia* “yuza 1” may maintain its essential photosynthesis for survival under severe drought stress.

## 5. Conclusion

In this study, a total of 107 miRNAs were identified by sRNA sequencing and 77 of them were differentially expressed (60 downregulated and 17 upregulated). 42 putative target genes were identified by degradome sequencing. The predicted target genes were annotated using the GO and KEGG databases. Functional analyses of the differentially expressed miRNAs and their corresponding targets revealed genes associated with response to stimulus, positive regulation of biological process, and transporter activity, which suggested that osmotic adjustment, plant hormone transduction, and synthesis of biomass played key roles in the response of *Paulownia* “yuza 1” to drought stress. Interestingly, we found that the novel miRNAs ptf-M1358 and ptf-M2218 may play important roles in the response to drought stress through affecting proteins related to photosynthesis. Besides, novel miRNAs ptf-M24a and ptf-M24b may also play a key role in the resistance to drought stress. The results of this study provided an insight into the molecular mechanisms of how *Paulownia* tree fights drought stress which can contribute to improve tolerance of this tree in future.

## Supplementary Material

The information of supplementary materials are as follows: Table S1: MiRNA qRT-PCR primers for *Paulownia* ‘yuza 1' PTF2W (control) and PTF2T (sever stress). Table S2: QRT-PCR primers of miRNA targets in *Paulownia* ‘yuza 1' PTF2W (control) and PTF2T (sever stress). Table S3: Sequences of miRNAs identified from *Paulownia* ‘yuza 1' PTF2W (control) and PTF2T (sever stress). Table S4: MiRNAs expression in *Paulownia* ‘yuza 1' PTF2W (control) and PTF2T (sever stress). Table S5: Putative miRNA targets identified by degradome sequencing in *Paulownia* ‘yuza 1' PTF2W (control) and PTF2T (sever stress).

## Figures and Tables

**Table 1:** Categories and statistical analysis of sRNAs in *Paulownia* “yuza 1” PTF2W (control) and PTF2T (severe stress).

Category	PTF2W				PTF2T			
	Unique sRNAs	Percent	Total sRNAs	Percent	Unique sRNAs	Percent	Total sRNAs	Percent
Total	3,221,622	100%	11,262,450	100%	3,177,839	100%	11,777,552	100%
miRNA	49,384	1.53%	2,593,930	23.03%	36,471	1.15%	4,086,959	34.70%
rRNA	39,391	1.22%	355,152	3.15%	42,204	1.33%	420,074	3.57%
snRNA	1256	0.04%	2508	0.02%	1288	0.04%	2723	0.02%
snoRNA	484	0.02%	1061	0.01%	441	0.01%	686	0.01%
tRNA	8277	0.26%	1,561,078	13.86%	8173	0.26%	448,506	3.81%
unann	3,122,830	96.93%	6,748,721	59.92%	3,089,262	97.21%	6,818,604	57.89%

**Table 2:** Statistical analysis of degradome data in *Paulownia* “yuza 1” PTF2W (control) and PTF2T (severe stress).

Category	PTF2W				PTF2T			
	Unique tags	Percent	Total tags	Percent	Unique tags	Percent	Total tags	Percent
rRNA	8193	0.09%	73,628	0.32%	10,416	0.09%	59,240	0.22%
tRNA	2213	0.02%	12,546	0.06%	2123	0.02%	8211	0.03%
snRNA	5867	0.06%	12,247	0.05%	8846	0.08%	18,564	0.07%
snoRNA	4519	0.05%	12,313	0.05%	6430	0.06%	19,028	0.07%
Poly (N)	13,943	0.15%	22,872	0.10%	19,312	0.18%	39,525	0.15%
cDNA_sense	2,926,724	32.17%	8,086,336	35.46%	3,496,643	31.83%	9,535,686	36.11%
cDNA_antisense	3,146,670	34.59%	9,168,559	40.21%	3,622,646	32.98%	10,214,034	38.68%
Other	2,988,689	32.85%	5,414,596	23.75%	3,817,634	34.76%	6,510,491	24.66%
Total	9,096,818	100.00%	22,803,097	100.00%	10,984,050	100.00%	26,404,779	100.00%
